# Supplementary material for: New Insights into the Sex Chromosome Evolution of the Common Barker Frog Species Complex (Anura, Leptodactylidae) Inferred from Its Satellite DNA Content
Source: Biomolecules. 2025 Jun 16;15(6):876. doi: 10.3390/biom15060876 (PMC12191414; doi:10.3390/biom15060876)
Supplement: Supplementary file 1 [file biomolecules-15-00876-s001.zip › Supplementary Figures and Tables.pdf]

Supplementary Figures

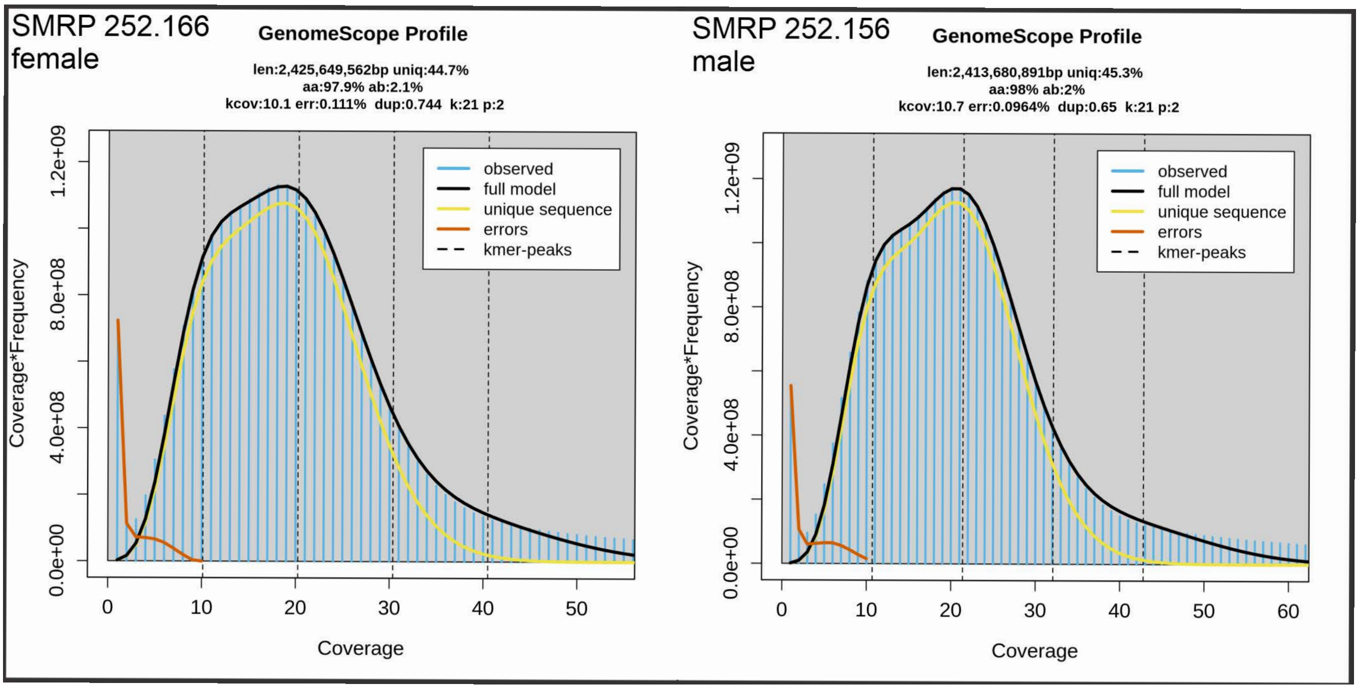

Supplementary Figure S1. Genome size estimation generated by GenomeScope2 for both female (left) and male (right) individuals of Ph. ephippifer, exhibiting genome sizes of 2.41 Gb and 2.43 Gb, respectively.

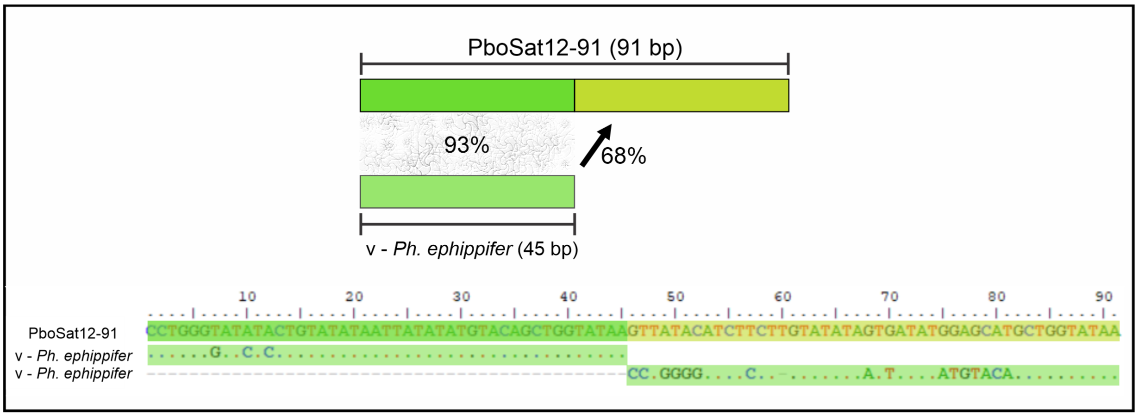

Supplementary Figure S2. Comparison between different variants from the same satDNA family observed in *Physalaemus ephippifer* and *Proceratophrys boiei*. Monomer of PboSat12-91 (MN475847.1) from *Pr. boiei*, with 91 bp, and variant from *Ph. ephippifer* with 45 bp. Note that the 45 bp monomer aligns with two regions of the *Pr. boiei* variant.

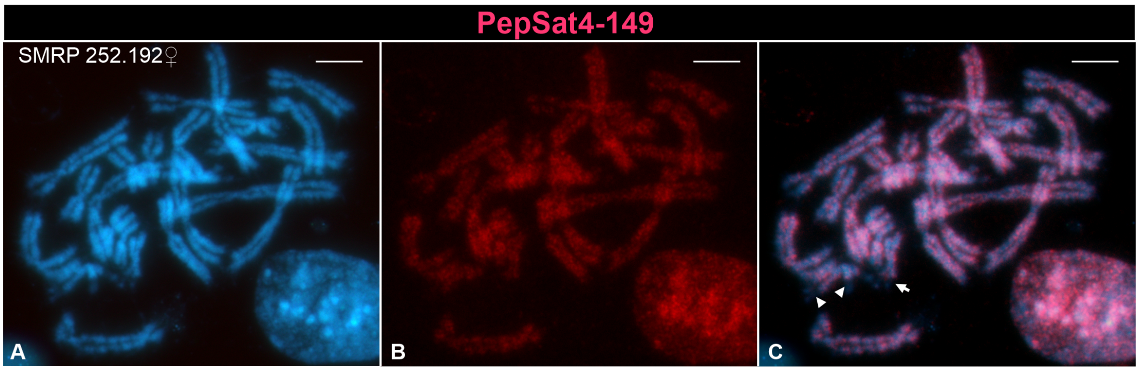

Supplementary Figure S3. Female karyotype of *Physalaemus ephippifer* from Santa Bárbara, Pará, Brazil, hybridized with the PepSat4-149 probe. Note the dispersed signal of the probe across all chromosomes, except at the NORs. **A.** DAPI staining. **B.** Probe signal. **C.** Merged images of **A** and **B**. The arrow indicates the NOR on the Z chromosome, while the arrowheads point to the NORs on the W chromosome. Scale bar = 5 µm.

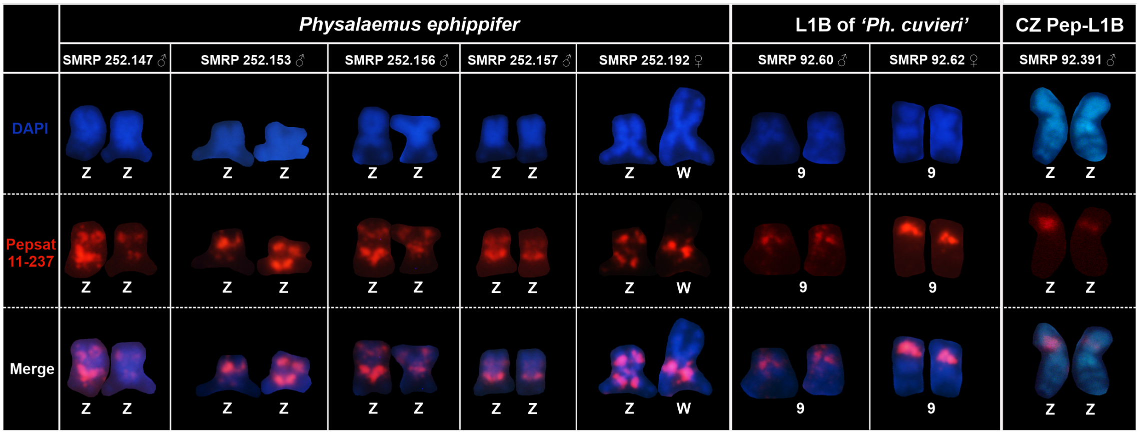

Supplementary Figure S4. Chromosome mapping of the PepSat11-237 probe in different specimens of *Physalaemus ephippifer*, lineage 1B (L1B) of *'Ph. cuvieri'*, and CZ Pep-L1B. Note the intra- and interindividual variation in probe intensity.

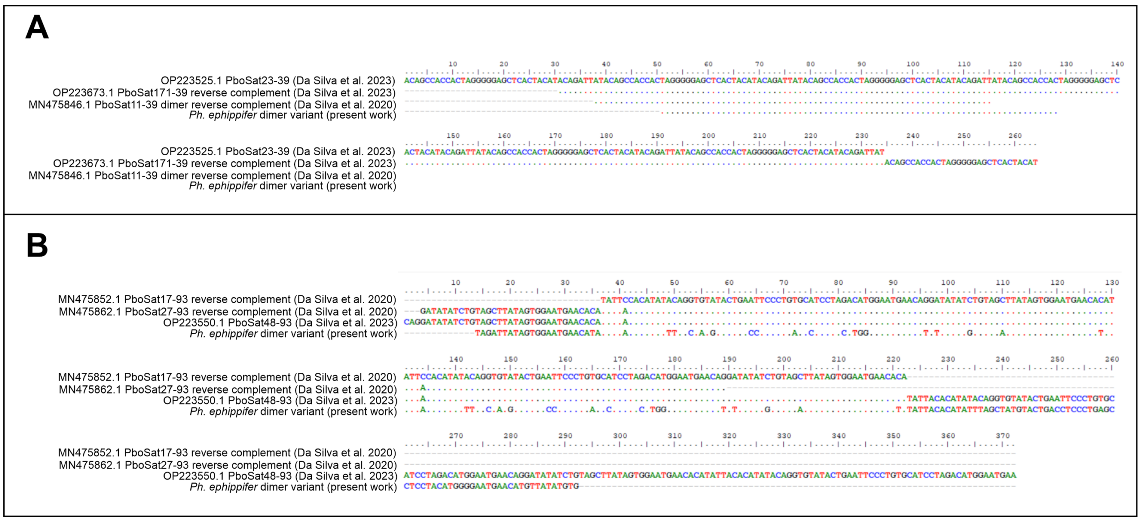

Supplementary Figure S5. Sequence alignment of two satDNA families with multiple descriptions (within the same study and across different studies), showing some similarity to *Physalaemus ephippifer* satDNAs. **A.** PboSat11-39. **B.** PboSat17-93.

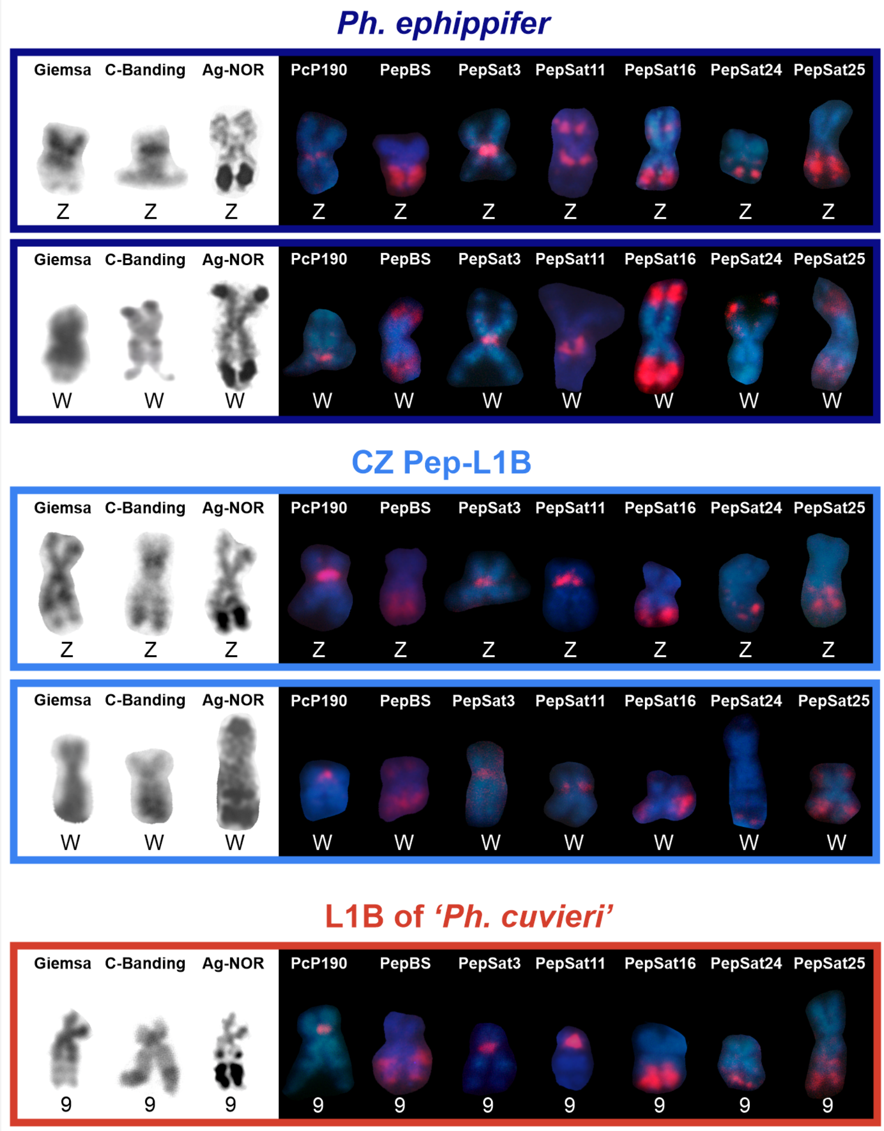

Supplementary Figure S6. Summary of the main chromosomal markers identified to date on the sex chromosomes of *Physalaemus ephippifer*, CZ Pep-L1B, and the homologous chromosome 9 of L1B of *'Ph. cuvieri'*.

Supplementary Tables

Supplementary Table S1. Primers used to amplify satellite DNAs by PCR.

satDNA	Primer	Sequence (5'-3')	Annealing temperature	Product size (bp)
Pepsat3-152	Pepsat3-F<br>Pepsat3-R	CATTGCAACGTTCCCAAGAT<br>TCATGCAGAATAGGCTTAATGG	57°C	140
Pepsat4-149	Pepsat4-F<br>Pepsat4-R	GGTATTAGGGTTACGCAGAGT<br>CCTTCACACAGGTTAAGCAC	54°C	151
Pepsat11-237	Pepsat11-F<br>Pepsat11-R	CAAACTCATTCTCGCGCTTA<br>CCGATGTCCCATTTGAACTC	57°C	219
Pepsat16-147	Pepsat16-F<br>Pepsat16-R	GGTGTGCTCATAAAACTGGC<br>GTAGCTGACAGACACTATGGA	54°C	144
Pepsat24-620	Pepsat24-F<br>Pepsat24-R	CCTAAGCCGATTGAAGACCT<br>CTGTTGCTTCCCACAAAACA	54°C	603
Pepsat25-282	Pepsat25-F<br>Pepsat25-R	ATGGGAATCTCATATGGCCG<br>ACCTCGATGAACAAGACACA	61°C	269
PepSat30-350	Pepsat30-F<br>Pepsat30-R	CCCTAACCCTAGCTCCATTC<br>AGACATCAGGGGAAGAGTTG	60°C	339

Supplementary Table S2. Abundance and divergence of the satellite DNAs (satDNAs) in male and female *Physalaemus ephippifer*. Divergence was calculated using the Kimura 2-Parameter model. Female-to-male (F/M) abundance ratios are also shown. *satDNAs with a ratio greater than 1.5. **satDNAs with a ratio lower than 0.75.

**satDNA**	**Abundance% (Divergence%)**		**F/M abundance ratio**
	**male**	**female**	
PepSat1-21	1.58 (10.75)	1.49 (10.35)	0.94
PepSat2-97	0.93 (7.63)	0.99 (7.50)	1.06
PepSat3-152	0.88 (1.52)	0.81 (1.42)	0.92
PepSat4-149	0.73 (9.72)	0.70 (9.59)	0.96
PepSat5-1310	0.46 (18.38)	0.44 (17.91)	0.94
PepSat6-1546	0.42 (11.04)	0.34 (11.13)	0.81
PepSat7-902	0.34 (14.12)	0.36 (14.04)	1.06
PepSat8-36	0.28 (11.78)	0.27 (11.82)	0.97
PepSat9-49	0.25 (20.14)	0.27 (19.49)	1.08
PboSat8-92-v-Pep	0.24 (11.11)	0.24 (10.95)	0.99
PepSat11-237**	0.27 (8.22)	0.20 (8.42)	0.73
PepSat12-73	0.24 (11.37)	0.23 (11.11)	0.98
PepSat13-49	0.22 (7.84)	0.21 (7.89)	0.97
PepSat14-100	0.21 (5.57)	0.23 (5.67)	1.08
PboSat05-35-v-Pep	0.19 (3.66)	0.19 (3.67)	0.96
PepSat16-147*	0.14 (8.44)	0.24 (8.75)	1.69
PepSat17-162	0.18 (11.91)	0.19 (12.05)	1.01
PepSat18-1073	0.17 (3.66)	0.18 (5.08)	1.06
PepSat19-629	0.16 (8.07)	0.17 (8.08)	1.03
PepSat20-22*	0.19 (10.84)	0.33 (8.27)	1.7
PepSat21-49	0.14 (18.59)	0.14 (18.10)	1.04
PcP190 1a	0.13 (1.47)	0.14 (2.49)	1.07
PboSat36-39-v-Pep	0.13 (10.33)	0.12 (10.32)	0.97
PepSat24-620	0.11 (9.13)	0.12 (7.84)	1.06
PepSat25-282*	0.06 (4.40)	0.15 (4.53)	2.24
PepSat26-908	0.09 (10.92)	0.09 (11.11)	0.98
PepSat27-280	0.09 (2.53)	0.09 (2.31)	1
PboSat22-90-v-Pep	0.08 (6.0)	0.09 (6.05)	1.12
PboSat11-39-v-Pep	0.07 (3.70)	0.07 (3.88)	1
PepSat30-350*	0.04 (5.65)	0.11 (3.83)	2.38
PepSat31-709	0.06 (6.28)	0.05 (6.83)	0.92
BBR86 v-Pep	0.05 (8.98)	0.05 (9.0)	1.04
PepSat33-30	0.04 (16.37)	0.04 (15.69)	1.01
PepSat34-57	0.04 (20.64)	0.04 (19.98)	0.99
PepSat35-28	0.05 (14.79)	0.04 (14.23)	0.81
PepSat36-739	0.04 (5.40)	0.03 (6.32)	0.77
PepSat37-101	0.04 (10.72)	0.04 (10.02)	0.97
PepSat38-39	0.03 (5.62)	0.04 (5.77)	1.13
PepSat39-90	0.03 (3.66)	0.03 (3.47)	0.98
PboSat12-91-v-Pep	0.03 (4.83)	0.03 (4.26)	1.01
PboSat17-93-v-Pep	0.02 (6.45)	0.02 (5.98)	1.09
PepSat42-41	0.02 (9.65)	0.02 (10.54)	0.99
PepSat43-54	0.02 (7.62)	0.02 (7.68)	1.3
PepSat44-16	0.02 (13.98)	0.02 (13.81)	1.05
PepSat45-33*	0.01 (5.33)	0.02 (3.04)	1.93
PepSat46-47	0.01 (10.99)	0.01 (11.18)	0.91
PepSat47-74	0.01 (3.04)	0.01 (2.91)	1.05
PepSat48-27	0.01 (4.25)	0.01 (4.29)	1.1
PepSat49-68	0.02 (6.08)	0.01 (6.12)	0.89
PepSat50-32**	0.02 (5.6)	0.008 (5.4)	0.35

PepSat51-109**	0.01 (14.27)	0.01 (12.03)	0.72
PepSat52-30	0.01 (6.15)	0.01 (6.02)	1.04
PepSat53-643*	0.01 (2.49)	0.01 (2.47)	1.54
PepSat54-31	0.01 (11.38)	0.01 (11.31)	1.19
PepSat55-33	0.01 (17.5)	0.01 (17.66)	0.89
PepSat56-31**	0.01 (5.41)	0.006 (7.25)	0.53
PepSat57-158*	0.006 (8.35)	0.01 (9.35)	1.68
PepSat58-45	0.009 (3.85)	0.009 (4.06)	0.94
PepSat59-30	0.009 (6.25)	0.009 (6.1)	0.96
PepSat60-110**	0.01 (5.99)	0.007 (6.6)	0.69
PepSat61-23	0.007 (5.21)	0.008 (4.97)	1.07
PepSat62-56**	0.007 (3.39)	0.0002 (17.38)	0.03
