# Supplementary material for: New Insights into the Sex Chromosome Evolution of the Common Barker Frog Species Complex (Anura, Leptodactylidae) Inferred from Its Satellite DNA Content
Source: Biomolecules. 2025 Jun 16;15(6):876. doi: 10.3390/biom15060876 (PMC12191414; doi:10.3390/biom15060876)
Supplement: Supplementary file 1 [file biomolecules-15-00876-s001.zip › Supplementary File S1.pdf]

Supplementary File S1 - Analysis of variant repeats of satDNA families found in *Physalaemus ephippifer*

Among the 62 satDNA families identified in *Physalaemus ephippifer* in this study, we analyzed all contigs generated by the RepeatExplorer pipeline to identify well-established variants in the *Ph. ephippifer* genome and assess the potential presence of a sex-linked variant. Each RepeatExplorer cluster is considered a distinct satellite DNA (satDNA) family. In addition to generating the most representative consensus sequence, the pipeline also provides contigs derived from the TAREAN analysis based on k-mer, which may exhibit distinct monomer compositions [35,36]. In our dataset, nine satDNA families with multiple contigs with similar k-mer coverage values or exceeding 0.5 were observed (Table 1). In this file, we present the key results and discussions we accessed through the analysis of these variants.

Table S1. List of satellite DNA families with well-established variants and key information about each variant. For variants with globally corresponding repeat units, the similarity percentage between them is shown. *Variants that did not show global alignment.

SatDNA family	Variant	Size (bp)	Similarity between variants (%)	GenBank accession number
PepSat1	v-a	21	81	PV463924
	v-b	21		PV463986
PepSat4	v-a	149	90	PV463927
	v-b	150		PV463987
PepSat5	v-a	1310	*	PV463928
	v-b	921		PV463988
PepSat17	v-a	162	*	PV463940
	v-b	72		PV463989
PboSat36-39	v-Pep-a	39	*	PV463946
	v-Pep-b	73		PV463990
PepSat36	v-a	739	*	PV463959
	v-b	618		PV463991
PepSat39	v-a	90	*	PV463962
	v-b	78		PV463992
PepSat45	v-a	33	*	PV463968
	v-b	38		PV463993
PepSat57	v-a	158	*	PV463980
	v-b	190		PV463994

Seven out of the nine satDNA families pointed out contained variants whose repeat units exhibited only partial correspondence and varied in length (i.e., PepSat5, PepSat17, PboSat36-v-Pep, PepSat36, PepSat39, PepSat45, and PepSat57). In the remaining two satDNA families with distinct variants (i.e., PepSat1 and PepSat4), the differences were primarily characterized by variations in nucleotide composition.

In the case of PepSat5, variants shared a 931 bp fragment, with the difference arising from an additional 389 bp region in v-a, which exhibits 61% similarity to its

adjacent region, likely originating from an ancient duplication (Figure 1A). In the remaining cases of variants with length differences, the most common pattern involved segment insertions and/or deletions. For example, in PepSat17, variants v-a and v-b contained 162 bp and 72 bp monomers, respectively, with a 72 bp region being 100% identical between them (Figure 1B).

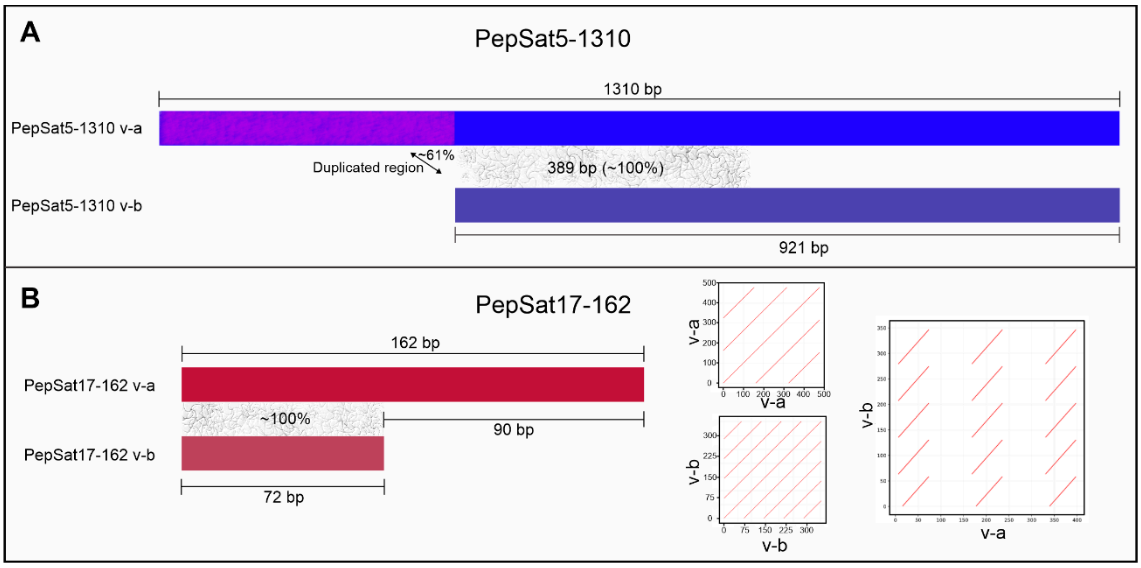

Figure S1. Representative diagrams of the satDNA families PepSat5-1310 (A) and PepSat17-162 (B) illustrating different variants. In A, the segment that distinguishes variants v-a and v-b likely originated from a duplication event, as the v-a region absent in the v-b can be aligned with the adjacent 389 bp sequence, displaying 61% similarity. However, in B, the 90 bp divergent fragment between the variants shows no similarity to any other part of the monomer.

Other distinctive and intriguing arrangements of variant repeats were observed in PepSat1 and PboSat36-39-vPep. Using dimers in RepeatProfiler with the combinations vava, vavb, and vbvb, we identified differential tandem organization in these cases. PepSat1 exhibited two variants of 21 bp each, with the most common pattern being an intercalated vavb arrangement (forming a 42 bp unit), while vava was also present. In contrast, vbvb was nearly absent (Figure 2A). Regarding the PboSat36-39, described by Da Silva et al. [51], it exhibited two variants in *Ph. ephippifer*: v-Pep-a, with 39 bp, and v-Pep-b, with 73 bp. The composition of v-Pep-b is nearly equivalent to two copies of the 39 bp unit found in v-Pep-a, differing only by a 5 bp deletion. However, when searching for the isolated 34 bp monomer, it was always found arranged composing a "dimer" together with the 39 bp unit (Figure 2B).

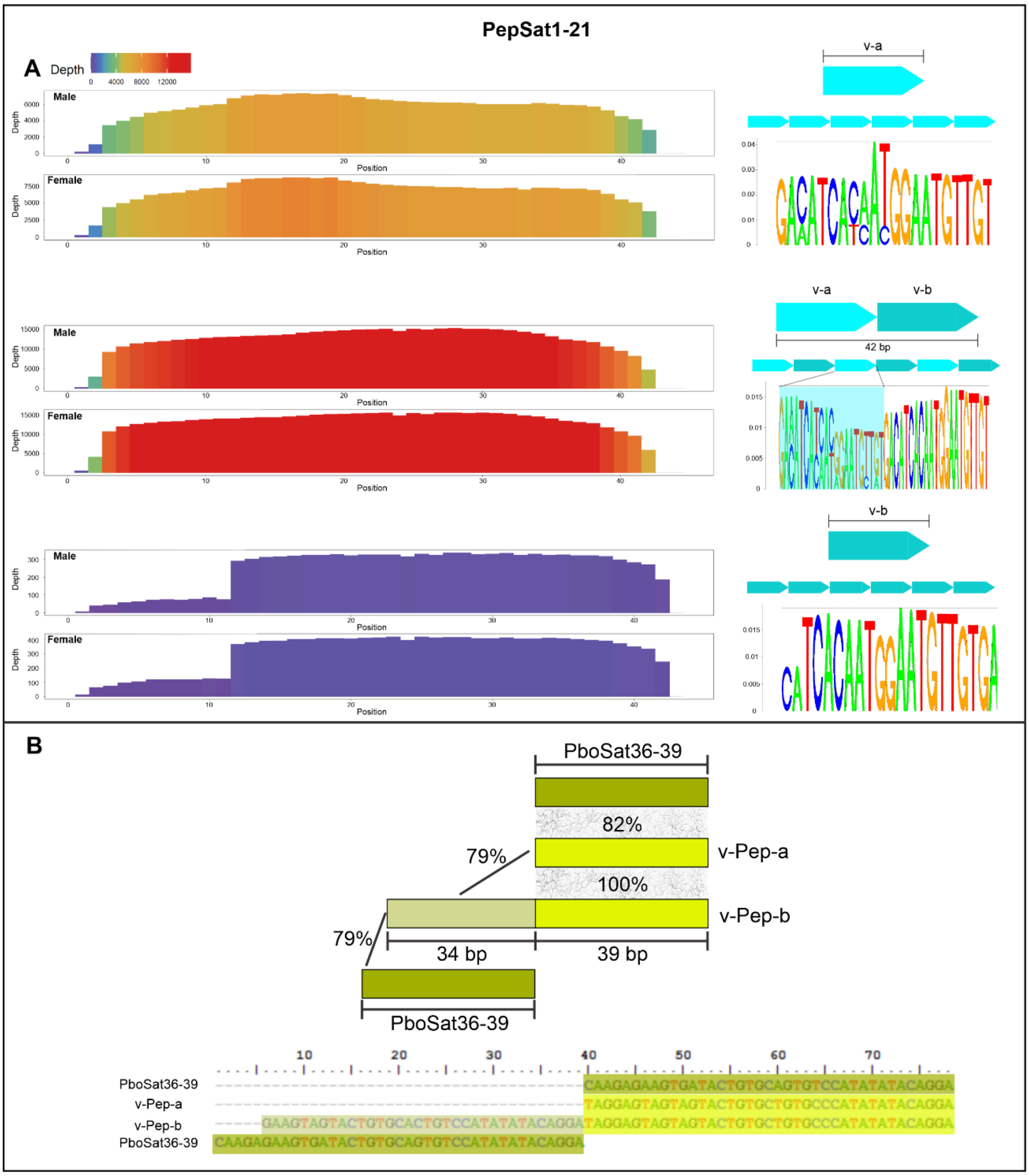

Figure S2. Schematic representation of the variants of PepSat1-21 (A) and PboSat36-39 (B), illustrating the different arrangements of these repeats within the genome. The depth and de Bruijn graphs shown in (A) were generated using RepeatProfiler (Negm et al. 2021) and RepeatExplorer (Novák et al. 2020), respectively. In (B), in addition to the variants present in *Physalaemus ephippifer*, we present a comparison with PboSat36-39, previously found in *Proceratophrys boiei* (Da Silva et al. 2023).

Both cases fit within a broader scenario in which new satDNAs arise from pre-existing ones through mutations and genetic drift, leading to the fixation of novel sequences (Figure 3). In this process, monomers may gradually diverge, forming

higher-order repeats (HORs) composed of multiple basic units, as observed in PepSat1. As a result of unequal crossing-over and the accumulation of mutations, these satDNAs may eventually lose their original smaller subunits in certain lineages, giving rise to new satDNAs with larger monomers, often doubling in size — a process hypothesized to have occurred in PboSat36-39-v-Pep-b. Conversely, the reverse process may also take place, with monomers becoming homogenized again through concerted evolution, which is known as a common process in the evolution of tandemly repeated sequences [2,4,6,7].

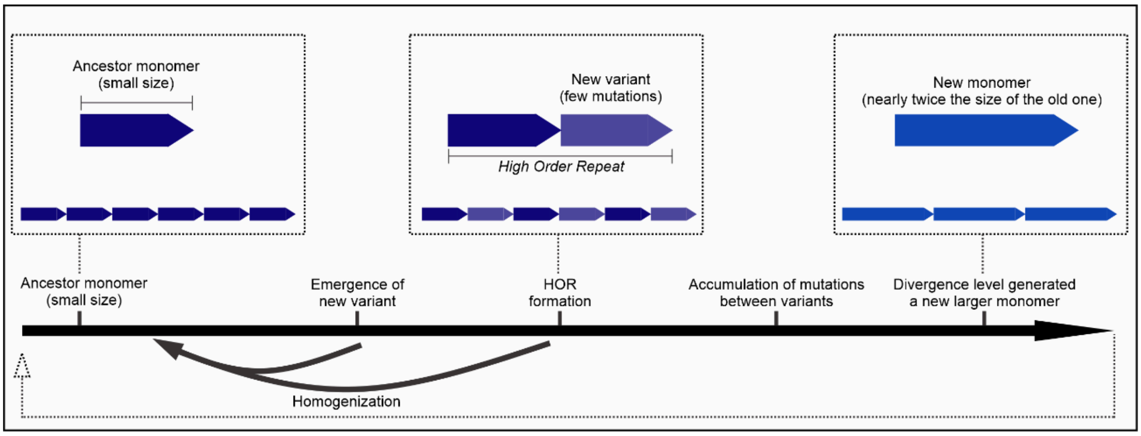

Figure S3. Schematic representation of a possible mechanism for the origin of larger satellite DNAs from smaller satDNAs. The process begins with the formation of a high-order repeat, which, through mutations and genetic drift, can diverge into a new sequence. Alternatively, homogenization may occur during this process.

In the remaining satDNAs with variants, we did not find the configuration in which both variants (v-a and v-b) are organized as HORs, except for PepSat5, where this arrangement was observed but only in the male sample. This result would suggest a sex-linked organization of this satDNA if *P. ephippifer* had male heterogamety. As this is not the case, this hypothesis is unlikely, as all chromosomes present in males are also found in females of this species. In this case, the most probable explanation is intrapopulational variation, similar to what was found for PepSat30, which was more abundant in the sequenced male genome due to the presence of an additional cluster of sequences on chromosome 5, as revealed by FISH (see Supplementary Figure S4).

Furthermore, PepSat45 and PepSat57 were the only satDNA families with a female-biased abundance (see Supplementary Table S2) and well-established distinct variants. In both satDNA families, v-a and v-b were more abundant in the female

sample. The most pronounced difference was observed in v-b of PepSat45, in which the read depth in RepeatProfiler mapping was approximately three times higher in the sequenced female genome than in the male sample, while for v-a, it was about twice as high (Figure 4A). A significant difference was also observed for PepSat57 variants, however, as this satDNA family is among the least abundant, the overall read depth remained low (Figure 4B). Additionally, although v-b of PepSat57 did not exhibit a substantial depth difference between the sexes, it was the only case in which certain SNPs were more represented in the female than in the male sample (Figure 4B).

Still regarding differences noted between the female and male samples, we can highlight the case of the variant v-Pep-a of PboSat36-39. Although no sex-linked difference in abundance was detected in the analysis of ratio conducted with RepeatMasker, the analysis of RepeatProfiler, performed with the separated variants, showed that the variant v-Pep-a of PboSat36-39 was more represented in the female sample than in the male sample (Figure 4C). Thus, at least in *P. ephippifer*, the PboSat36-39 v-Pep-a may represent a candidate sex-linked variant, whereas PboSat36-39 v-Pep-b does not. This result was likely masked in the RepeatMasker analysis because only the most common consensus sequence per family was used, and only one of the two variants within this family appeared to be sex-linked.

These data on candidate sex-linked variants are particularly interesting, as they suggest that these satDNAs may be present on the sex chromosomes. With the reduction of recombination between the Z and W chromosomes, distinct variants may have become fixed in these different chromosomes. The accumulation of mutations and sequence divergence are expected to occur more frequently on the W chromosome, whereas the Z chromosome, present in two copies in males, likely undergoes more frequent recombination, as revised in Garrido-Ramos [4]. Nonetheless, these findings need to be corroborated through additional approaches, as the observed variation could result from a bias in the sample used for genome sequencing.

Figure S4. Depth profile differences between sexes for variants of PepSat45 (A), PepSat57 (B), and PboSat36-39-v-Pep-a (C). Note that variants in these three satDNA families exhibit higher abundance in the female sample. In (C), an additional summary highlights v-b of PepSat57 at base-pair resolution, with asterisks indicating nucleotides that show differential distribution between sexes. All figures were generated using RepeatProfiler (Negm et al. 2021).

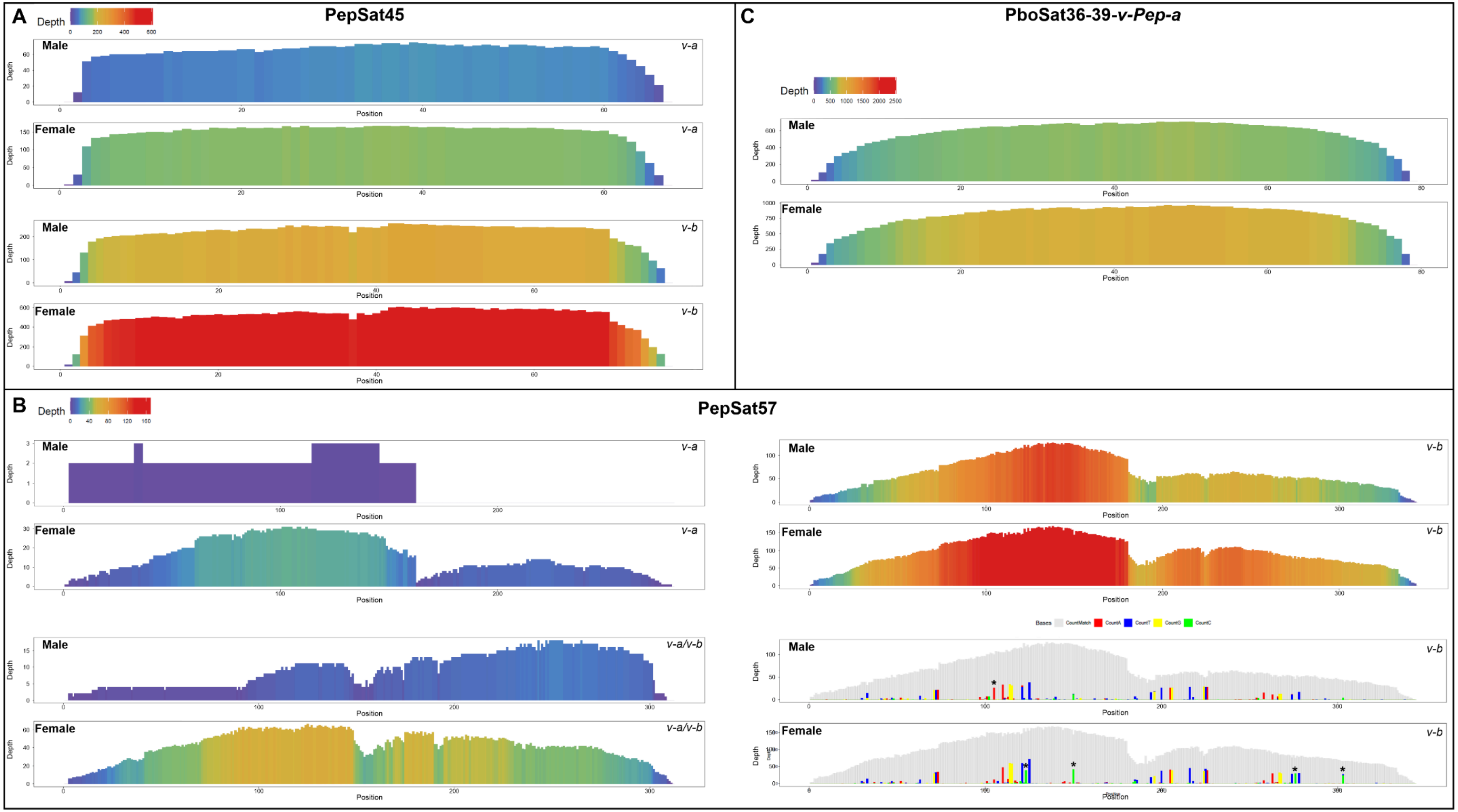
A
Depth
PepSat45
Male
v-a
Female
v-a
Male
v-b
Female
v-b
Position
C
PboSat36-39-v-Pep-a
Depth
Male
Female
Position
B
Depth
PepSat57
Male
v-a
Female
v-a
Male
v-a/v-b
Female
v-a/v-b
Male
v-b
Female
v-b
Bases
CountMatch
CountA
CountT
CountG
CountC
Male
v-b
Female
v-b
Position
